# Pseudo-pseudo Meigs’ syndrome presenting with a combination of polyserositis, elevated serum CA 125 in systemic lupus erythematosus: A case report

**DOI:** 10.1097/MD.0000000000015393

**Published:** 2019-04-26

**Authors:** Fei Gao, YongMei Xu, GuoWang Yang

**Affiliations:** Department of Hematology & Oncology, Beijing Hospital of Traditional Chinese Medicine, Clinical Medical College of Traditional Chinese Medicine, Capital Medical University, Beijing, China.

**Keywords:** CA 125, polyserositis, pseudo-pseudo Meigs’ syndrome, systemic lupus erythematosus

## Abstract

**Rationale:**

Combination of polyserositis and elevated serum CA 125 is common in tumor or infectious disease, but this clinical combination is also found in other diseases. It could be the initial manifestation of pseudo-pseudo Meigs’ syndrome (PPMS), which is characterized by the presence of polyserositis and raised CA-125 level in systemic lupus erythematosus (SLE).

**Patient's concerns:**

A 44-year-old Chinese female was admitted with three months history of painless abdominal distension accompanied by watery diarrhea 5–6 times daily, shortness of breath, fatigue, lower limb swelling, and 10 kg weight loss. The test results showed peripheral cytopenias, hypoproteinemia, renal dysfunction and elevated CA 125, antidouble-stranded DNA antibodies, and anti-Sjogren's syndrome A antigen antibody was positive. There is no evidence for the diagnosis of solid tumor according to the results of imaging modality and pathological examination.

**Diagnosis:**

The patient was diagnosed as pseudo-pseudo Meigs syndrome.

**Intervention:**

The patient received hormone, leflunomide, and Plaquenil therapy.

**Outcomes:**

The patient's symptoms were relieved and the laboratory index was improved after the treatment of hormone and immunosuppressant.

**Lessons subsections as per style:**

PPMS is characterized by the combination of serous effusion and elevated serum CA 125 with no evidence of tumor among SLE patients. Clinicians should be aware of the diagnosis of PPMS avoiding unnecessary anxiety or surgical interventions.

## 1 Introduction

The clinical manifestations of polyserous effusions (such as pleural effusion, ascites, etc.) combined with elevated serum CA 125 often occur in tumor disease, but this clinical combination is also found in nontumor patients, such as tuberculosis, nephrotic syndrome, connective tissue disease, and so on. Systemic lupus erythematosus (SLE) is an autoimmune disease characterized by multiple autoantibodies and multisystem involvement. We reported the case of SLE patient presented with pseudo-pseudo Meigs’ syndrome-polyserositis (PPMS) and elevated serum CA 125. PPMS was first reported by Tjalma.^[1]^ At present, there is no definite conclusion on the pathogenesis of PPMS. Up to now, only more than 10 reports about PPMS have been published. To our knowledge, what we reported is the first case presenting with multiple system damage. Informed written consent was obtained from the patient for publication of this case report and accompanying images.

## 2 Case report

Beijing Hospital of Traditional Chinese Medicine, Clinical Medical College of Traditional Chinese Medicine, Capital Medical University Institutional Review Board approved the publication of this article. A 44-year-old Chinese female presented to our hospital in January 2018 with three months history of painless abdominal distension accompanied by watery diarrhea 5 to 6 times daily, shortness of breath, fatigue, lower limb swelling, and 10 kg weight loss without fever, oral ulcers, or Raynaud phenomenon. She denied history of connective tissue.

Physical examination on admission showed anemic appearance, low spirits, hair sparsely, and mild telangiectasia in the face. Her lower lung fields sounds were a bit quieter than normal, heart rate was 97 beats per minute with no significant pathological murmur, abdominal distension, shifting dullness positive, and slight pitting edema over both legs.

The results of admission examination were as follows: white blood cell (WBC) 2.87 × 10^9^/L, neutrophil% (NEUT%) 57.1%, hemoglobin (HgB) 79 g/L, platelet (PLT) 282 × 10^9^/L, albumin (ALB) 29 g/L (40–55), creatinine (Cr) 124 μmol/L (45–84), blood urea nitrogen (BUN) 4.79 mmol/L (3.3–7.5), uric acid (UA) 492 μmol/L (155–357). Hepatic function was normal, urine routine BLD2+, PRO2+. The 24 h urine protein quantitate was 1378.1 mg/24 h. TB infects T-cell spots (−).

Immunological index examination showed antinuclear antibody (ANA) 1:1000 (+), antidouble-stranded DNA antibodies (anti-dsDNA) 109.91 IU/ml, anti-Sjogren's syndrome A antigen antibody (anti-SSA) 114 (+++). C3 0.48 g/L (0.75–1.4), C4 0.09 g/L (0.1–0.4), C1q 125.13 mg/L (159–233).

Screening for tumor markers showed elevated serum CA 125 at 360.8 U/ml, Depth detection of supine ascites 9.7 cm. The appearance of ascites was yellow and transparency was cloudy. Total number of cells in ascites was 678 × 10^6^/L, WBC 38 × 10^6^/L. Gravity of ascites was 1.024. Multiple ascites pathology showed numerous lymphocytes, histiocytic, and mesothelial cells can be visible in ascites without tumor cells or acid-fast bacilli Rivalta (+). Ascites fluid was sent for tuberculosis polymerase chain reaction (TB-PCR) and the result was negative.

**Figure 1:**
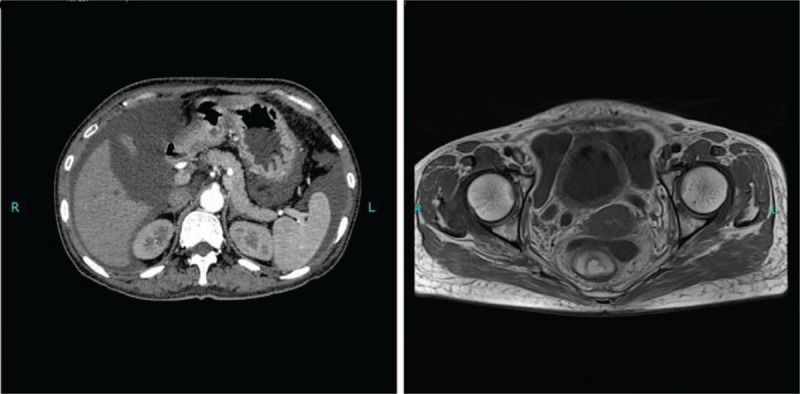
CT scan of the abdomen showing massive ascites, no tumor lesion found in CT scan of the abdomen and pelvic MRI. CT = computed tomography, MRI = magnetic resonance imaging.

**Figure 2:**
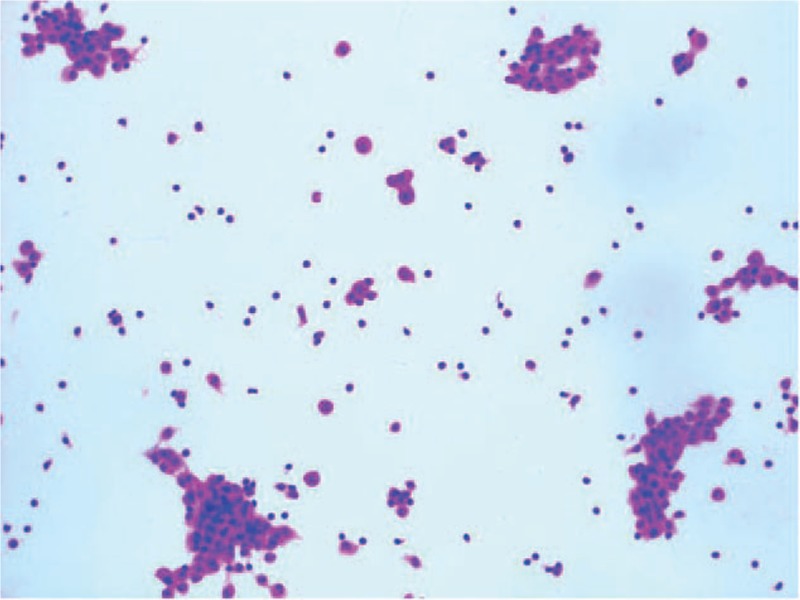
After extraction of ascites, pathology showing the ascitic fluid contains lymphocytes, histiocytes, normal mesothelial cells.

The chest computed tomography indicated pleural effusion. There is no evidence for the diagnosis of solid tumor (benign tumor or malignancy) according to the results of imaging modality (Fig. 1) and pathological examination (Fig. 2).

Clinical manifestations of this case include multiple serosa effusion and diarrhea, the test results showed peripheral cytopenias, renal dysfunction, elevated CA 125, anti-dsDNA, and anti-SSA positive. Eighty milligram per day of methylprednisolone therapy was given intravenously for 3 days, and then, the dosage was reduced to 40 mg/day and continued intravenously for 3 days, subsequently, 1 mg/kg/day prednisone acetate tablets were given orally as maintenance therapy, the prednisone acetate dosage was reduced by 2.5 mg weekly and maintained at a dose of 10 mg/day. Leflunomide was taken orally at 20 mg/day. Hydroxychloroquine Sulfate Tablets were taken orally at 400 mg/day. Two months after treatment with hormone, Leflunomide and Plaquenil, the clinical symptoms, and clinical test indexes of the patients were significantly improved. Depth detection of supine ascites was reduced to 2.9 cm, pleural effusion disappeared and defecation was normal. Examination at the time of revisit in April 2018 were WBC 4.7 × 10^9^/L, NEUT% 56%, HgB 84 g/L, PLT 290 × 10^9^/L, ALB 35.2 g/L, Cr 67.9 mmol/L, BUN 7.3 mmol/L, UA 340 mmol/L, serum CA 125: 23.57 U/ml and urine protein quantitate was 725.6 mg/24 h.

## 3 Discussion

According to the results of the patient's ascites examination, we judged that the ascites were effusion, and referred to patient admission examination, we excluded the pleural and ascites caused by cardiac insufficiency, nephrotic syndrome, and liver cirrhosis because they often form leaking fluid. Also, physico-chemical examination did not support the above diagnosis.

Tuberculosis, tumor, and connective tissue disease are the main reasons for the formation of exudate. No tumor cells were discovered after multi-pathological examination. No definite tumor lesions were found in Pelvic Magnetic Resonance Imaging and CT image of chest and abdomen. TB infects T-cell spots result and ascitic fluid for many times to find acid fast bacilli do not support tuberculosis diagnosis. This patient had pleural effusion, renal involvement (abnormal creatinine, hematuria, and proteinuria), hemocyte decrease, hypocomplementemia, and raised antibody titer (ANA cytoplasm 1:1000 [+], anti-dsDNA antibody: 109.91 IU/ml, anti-SSA 114 [+++]). According to the diagnostic criteria of SLE recommended by the European League Against Rheumatis (EULAR) and American College of Rheumatology (ACR) in 2007,^[2]^ the patient was diagnosed as SLE. However, combination of serum CA 125 elevation and multiple serous effusion is rare in SLE patients, which led us to a diagnosis PPMS.

PPMS is characterized by the combination of serous effusion and elevated serum CA 125 with no evidence of tumor among SLE patients.^[3]^ Different from Meigs’ syndrome, PPMS is unrelated to benign or malignant ovarian masses, Tjalma^[1]^ described this condition as Tjalma syndrome, which is identical to PPMS. Till now, only a few case reports about PPMS have been published. At present, the pathogenesis of PPMS is not clear, but it was showing that uncontrolled severe inflammation may be one of the causes of PPMS in lupus.^[4]^ Glucocorticoids are potent anti-inflammatory and immunosuppressive agent, which exert their effects by reducing the expression of cytokines and inhibiting almost all primary and secondary immune cells.^[5]^ The patient received prompt hormone therapy that resulted in stabilization of the lab values and symptoms improvement.

The tumor marker CA 125 is a glycoprotein expressed in the epithelial cells, which was detected in ovarian carcinoma cells using monoclonal antibody OC125 and proposed as a specific marker for ovarian carcinoma.^[6]^ Because the glycoprotein of CA 125 can be secreted by mesothelial cells of the peritoneum, pleura, and pericardium, epithelium of fallopian tubes, endometrium, endocervix, pericardium, lung, breast, etc.^[7,8]^ In other cases, such as pregnancy, menstruation, and ascites, CA 125 can also be higher than normal.^[9–12]^ There is more evidence to suggest that the rise of CA 125 is associated with SLE combined with tumor, nephrotic syndrome, or seroserotis.^[13,14]^ Elevated CA 125 levels in PPMS patients is considered to be a result of the interaction between cytokines (vascular endothelial growth factor, fibroblast growth factor) and mesothelial cells.^[15]^ In this case, we speculated the increased CA 125 serum levels are the result of a triggering of the mesothelial cells.

The decrease of serum C3 and C4 in this patient was related to the complement consumption caused by complement system activation, and the activated C3 and C4 are involved in the inflammatory reaction, which can induce autoantibodies to form immune complexes with their antigens and deposit in organs and tissues, resulting in damage to tissues and organs.^[16]^ In this case, SLE involvement in the blood system and kidneys is the cause of hemocyte depletion and renal dysfunction. ALB is a plasma protein and carrier protein. The inflammatory reaction of SLE can cause ALB overdegradation. In addition, the activation of the complement increases the capillary permeability and causes protein loss and which leads to a significant decrease in the serum ALB level.^[17,18]^ We speculated the reason for this patient's diarrhea is due to intestinal damage caused by SLE, resulting in protein-losing enteropathy (diarrhea, bowel wall thickening, and mesenteric structural disorder), which needed to be confirmed by 99mTc-HAS (99m-labeled human serum albumin)^[19]^ or alpha-1-antitrypsin clearance instool examination,^[20]^ but which are not available at our hospital.

We have summarized the diagnostic criteria for PPMS in literature. The diagnosis of PPMS needs to meet the following three conditions: (1) painless, gradual onset polyserositis, and elevated CA 125 in SLE patient; (2) either benign or malignant tumors are excluded; (3) polyserositis due to pathologic factors by SLE (such as lupus nephritis complicated by nephrotic syndrome and lupus peritonitis) are excluded.

According to the diagnostic criteria of SLE recommended by EULAR and ACR in 2007,^[2]^ the patient was diagnosed with SLE. Furthermore, presences of SLE, polyserous effusions and high serum CA 125 levels led us to a diagnosis of PPMS.

For this patient, conservative treatment was used in the treatment and it produced a good curative effect avoiding unnecessary anxiety or surgical interventions. So combination of polyserositis, elevated serum CA 125 does not mean the diagnosis of tumor or infectious diseases (such as tuberculosis). The clinical combination can be found in connective tissue disease. Clinicians should be aware of the diagnosis of PPMS when they have similar clinical manifestations excluding tumor diagnosis in the future.

## Author contributions

**Writing – original draft:** Fei Gao.

**Writing – review & editing:** YongMei Xu, GuoWang Yang.
